# The Positioning Accuracy of BAUV Using Fusion of Data from USBL System and Movement Parameters Measurements

**DOI:** 10.3390/s16081279

**Published:** 2016-08-15

**Authors:** Naus Krzysztof, Nowak Aleksander

**Affiliations:** 1 Faculty of Navigation and Naval Weapons, Polish Naval Academy, Smidowicza 69, Gdynia 81-103, Poland; 2 Faculty of Civil and Environmental Engineering, Department of Geodesy, Gdansk University of Technology, Narutowicza 11/12, Gdansk 80-233, Poland; aleksander.nowak@geodezja.pl

**Keywords:** underwater positioning, estimation of coordinates, autonomous biomimetic underwater vehicle

## Abstract

The article presents a study of the accuracy of estimating the position coordinates of BAUV (Biomimetic Autonomous Underwater Vehicle) by the extended Kalman filter (EKF) method. The fusion of movement parameters measurements and position coordinates fixes was applied. The movement parameters measurements are carried out by on-board navigation devices, while the position coordinates fixes are done by the USBL (Ultra Short Base Line) system. The problem of underwater positioning and the conceptual design of the BAUV navigation system constructed at the Naval Academy (Polish Naval Academy—PNA) are presented in the first part of the paper. The second part consists of description of the evaluation results of positioning accuracy, the genesis of the problem of selecting method for underwater positioning, and the mathematical description of the method of estimating the position coordinates using the EKF method by the fusion of measurements with on-board navigation and measurements obtained with the USBL system. The main part contains a description of experimental research. It consists of a simulation program of navigational parameter measurements carried out during the BAUV passage along the test section. Next, the article covers the determination of position coordinates on the basis of simulated parameters, using EKF and DR methods and the USBL system, which are then subjected to a comparative analysis of accuracy. The final part contains systemic conclusions justifying the desirability of applying the proposed fusion method of navigation parameters for the BAUV positioning.

## 1. Introduction

Determining the position coordinates with high accuracy during the process of conducting marine underwater navigation is becoming a significant problem. This is mainly due to the fact that the Global Navigational Satellite System (GNSS) cannot be used. Sometimes using satellite navigation during emersion could be also difficult, especially in port areas, where infrastructure facilities and other vessels can block and reflect navigation signals [1,2]. Due to these difficulties, it becomes necessary to use dedicated navigation systems (NS) in such cases [3,4,5,6,7,8,9,10]. These dedicated navigation systems generally:

- use devices to carry out navigational parameter measurements in water, such as log, hydrostatic pressure sensor, or echo-sounder,
- estimate the position coordinates as a result of the fusion of various navigational parameters, e.g., speed with course or submergence [11,12,13,14,15,16,17,18,19,20].

In the case of BAUV, a selection of devices and methods for determining the position can be made dependent on the parameters of its operation, shape, and size of the hull (construction), and type of propulsion used.

**Figure 1:**
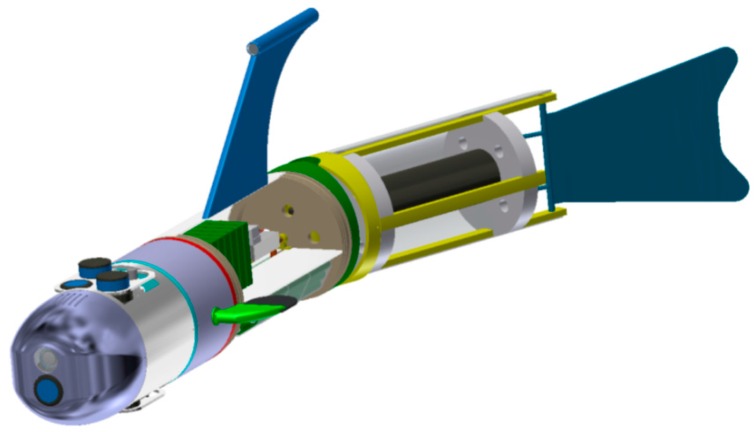
Side view of 3D model of the BAUV built in Polish Naval Academy (3D design by Bogdan Szturomski) [9].

A BAUV, imitating fish in terms of the design and movement, is currently being prepared at the Polish Naval Academy (PNA) (Figure 1).

Starting from the bow to the tail fin, the BAUV consists of the following modules [9]:

- module with camera and “looking” forward echo sounder (“wet” compartment),
- module of the sensors (in the upper part: USBL, hydro modem and “looking” up echo sounder; in the bottom part: sonar and “looking” down echo sounder—“wet” compartment),
- module of the lateral fins (“dry” compartment),
- module of electronics and batteries (the batteries located in the bottom part, which can move along the longitudinal axis of the vehicle giving the possibility of trimming; two computers PC-104 and power management system mounted in the upper part—the whole compartment is “dry”),
- module of the caudal fin consisting of two segments (the tail fin driven by an electric motor with nominal power 250 W, rotary motion converted into an oscillating motion).

The range of swimming is designed for about 2 nautical miles and assumed optimum cruise speed is around 1 m/s (2 knots). The swimming range is an important operating parameter and it is a determining factor in the construction of the designed NS.

Several NS variants have been designed for the conceptual stage of the BAUV. They differ from each other, mainly in applied navigation devices and in algorithms of the position coordinates estimation. The conceptual NS described in this publication consists of components installed on the BAUV:

- GPS Aided Inertial Navigation System (GPS/INS) “VN-200” [21]—due to the operating environment, it is not possible to use GPS, therefore this subsystem is referred to as INS “VN-200” in subsequent parts of this article,
- “ALIZE” electro-magnetic log [22],
- ”WIKA S-20” hydrostatic pressure sensor [23],
- USBL “MicronNav System” transponder system [24],
- “MICRON DATA MODEM” slave hydromodem [25],

and components located on the seabed:

- USBL “MicronNav System” transceiver system,
- “MICRON DATA MODEM” master.

The USBL system will be dedicated to measure the approximate position coordinates (sending to the BAUV by hydromodem). The INS “VN-200” will measure the course, while the log will measure speed, and the pressure sensor will be used to submergence determination. These parameters will then be used to estimate submerged BAUV position by the extended Kalman filter. The BAUV moves using wave motion.

The NS constructed in this way will be subject to a comparative evaluation of the dead reckoning NS (that uses only the course and speed measurement), and the NS based only on the USBL system. The basic criterion for this assessment is the accuracy of the estimated position coordinates.

## 2. Evaluation of the Accuracy of Determining the Position Coordinates Using the USBL System

**Figure 2:**
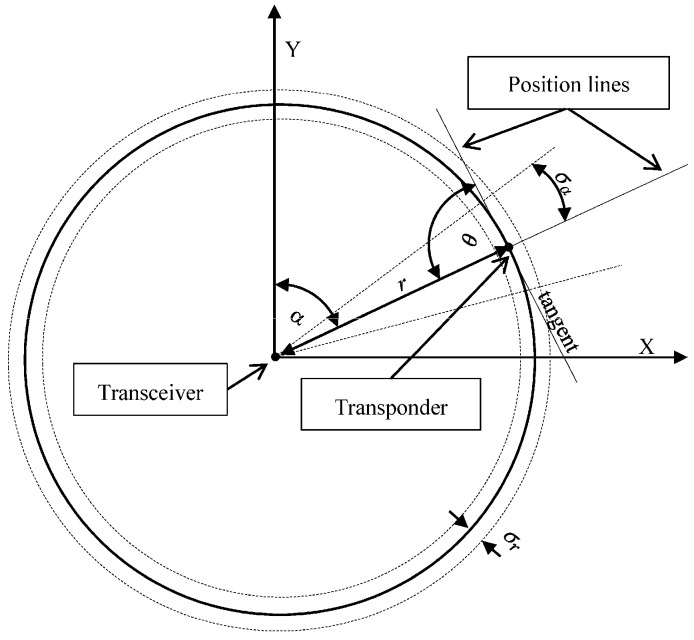
The principle of determining the horizontal coordinates (x, y) using USBL system.

Determining the horizontal coordinates using USBL system is based on the measurement of two parameters: direction *α* (yaw) and the distance between the Transceiver and the Transponder r (Figure 2).

The mean error $M_{xy}$ and the mean error ellipse (i.e., the length of the *a* and *b* axes) of determined horizontal coordinates (x, y) using the USBL system can be calculated by applying the law of mean errors propagation [26,27,28]. Knowing the functions of the individual test result:

(1) $$\alpha =\text{arc tg}\frac{x}{y}$$

(2) $$r=\;\sqrt{x^{2}+y^{2}\;}$$

the equations of position lines mean error can be represented by:

(3) $$\sigma_{l(\alpha )}=\sigma_{\alpha }/\sqrt{{\left(\frac{\partial \alpha }{\partial x}\right)}^{2}+{\left(\frac{\partial \alpha }{\partial y}\right)}^{2}}$$

(4) $$\sigma_{l(r)}=\sigma_{r}/\sqrt{{\left(\frac{\partial r}{\partial x}\right)}^{2}+{\left(\frac{\partial r}{\partial y}\right)}^{2}}$$

that enables determination of:

(5) $$M_{xy}=\frac{1}{\sin\theta }\sqrt{{\sigma_{l(\alpha )}}^{2}+{\sigma_{l(r)}}^{2}}=\sqrt{{\left(\sigma_{\alpha }\cdot r\right)}^{2}+{\sigma_{r}}^{2}}$$

(6) $$a=\sigma_{\alpha }\cdot r$$

(7) $$b=\sigma_{r}$$

where θ—the intersection angle of the position lines. In the case of USBL it always equals 90°; $\sigma_{\alpha }$—α (yaw) mean measurement error; $\sigma_{r}$—r (distance) mean measurement error; r—distance between the Transceiver and Transponder; a—the length of the major axis of the mean error ellipse; b—the length of the minor axis of the mean error ellipse.

**Figure 3:**
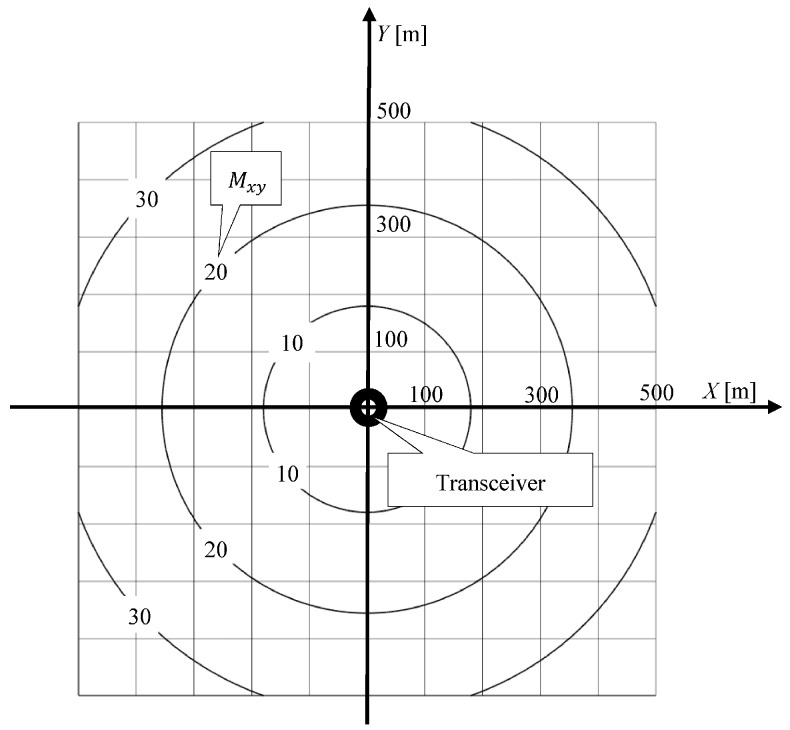
Mapping the accuracy of the coordinate position of determined USBL system (describes mean error $M_{xy}$ in meters).

Based on the dependence Equation (5), information provided by the manufacturer, and the values of the mean errors of measurement, $\sigma_{\alpha }=3{}^{\circ}$ i·$\sigma_{r}=0.2$ m, an area map has been compiled on the accuracy of position coordinates, determined by the Tritech USBL “MicronNav System” (Figure 3) [24].

Figure 3 shows that the mean errors of position coordinate increases with the measured distance. It reaches 26.2 m at a distance of 500 m. This is the distance limit at which the USBL “MicronNav System” can work [24].

**Figure 4:**
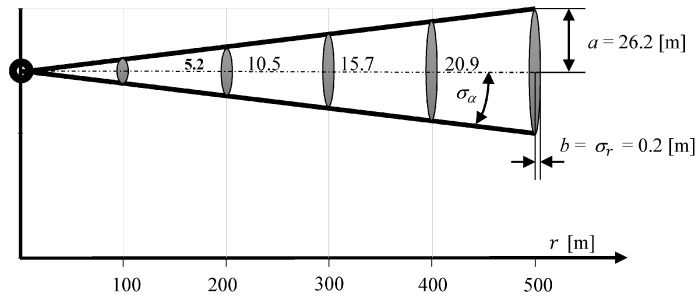
Changing the parameters of the mean error ellipse as a function of measured distance.

Figure 4 shows the mean error ellipse of position coordinates determined in the same direction, in one hundred meter intervals.

It shows clearly that the coordinate error increases substantially in a perpendicular direction to the measurement direction with increasing distance. However, it is constant in direction of the measurement being carried out, and is only 0.2 m.

## 3. Evaluating the Accuracy of Determining the Position Coordinates Using Dead Reckoning

If the impact of current, drift, and waving of sea on the BAUV movement can be avoided, the only sources of errors are the devices used to dead reckoning. In the case of the BAUV, these are the Inertial Navigation System, INS “VN-200”, which determines course over ground (COG) [21] and the electromagnetic log “ALIZE”, which determines speed through water (STW). In this case, the STW is equal to the speed over ground (SOG) [22].

By measuring using these devices COG(k), SOG(k) at the time of (k), vector $x_{h}$ of horizontal coordinates and their accuracy can be calculated for the time (k+1). Functions $f\left(x_{h}\left(k\right),u_{h}\left(k\right),w_{h}\left(k\right)\right)$ can be used for this purpose describing the non-linear BAUV movement model:

(8) $$x_{h}\left(k+1\right)\;=\left[\begin{array}{c} \begin{array}{c} f_{x}\left(k+1\right)\\ f_{y}\left(k+1\right) \end{array} \end{array}\right]=\left[\begin{array}{c} \begin{array}{c} x\left(k\right)\\ y\left(k\right) \end{array} \end{array}\right]+\left[\begin{array}{c} \begin{array}{c} \Delta t\left(k\right)\cdot \text{SOG}\left(k\right)\cdot \sin\text{COG}\left(k\right)\\ \Delta t\left(k\right)\cdot \text{SOG}\left(k\right)\cdot \cos\text{COG}\left(k\right) \end{array} \end{array}\right]+\left[\begin{array}{c} \begin{array}{c} w_{x}\left(k\right)\\ w_{y}\left(k\right) \end{array} \end{array}\right]$$

where x(k),y(k)—dead reckoned horizontal coordinates of BAUV position at the time of k; COG(k)—the BAUV course over ground at the time of k; SOG(k)—the BAUV speed over ground at the time of k; Δt(k)—duration between the time of *k* and *k* + 1; $w_{x}\left(k\right),w_{y}\left(k\right)$—the so-called intentional interference in determining the coordinates at the time of *k* (expressed as a zero mean normal distribution N[0, 1]); and calculated on the basis of Equation (8) the covariance matrix

(9) $$P\left(k+1\right)=F_{x}\left(k+1\right)P\left(k\right)F_{x}{\left(k+1\right)}^{T}+Q\left(k\right)$$

here: $F_{x}\left(k+1\right)=\left[\begin{array}{cc} \frac{\partial f_{x}}{\partial x} & \frac{\partial f_{x}}{\partial y}\\ \frac{\partial f_{y}}{\partial x} & \frac{\partial f_{y}}{\partial y} \end{array}\right]=\left[\begin{array}{cc} 1 & 0\\ 0 & 1 \end{array}\right]$—the so-called matrix system is calculated as Jacobian matrix from the function f(x(k),u(k),0); P(k)—the covariance matrix, determined at the time of k.

$$Q\left(k\right)=\left[\begin{array}{cc} \frac{\partial f_{x}}{\partial COG} & \frac{\partial f_{x}}{\partial \text{SOG}}\\ \frac{\partial f_{y}}{\partial \text{COG}} & \frac{\partial f_{y}}{\partial \text{SOG}} \end{array}\right]\left[\begin{array}{cc} {\sigma_{COG}}^{2} & 0\\ 0 & {\sigma_{SOG}}^{2} \end{array}\right]{\left[\begin{array}{cc} \frac{\partial f_{x}}{\partial COG} & \frac{\partial f_{x}}{\partial \text{SOG}}\\ \frac{\partial f_{y}}{\partial \text{COG}} & \frac{\partial f_{y}}{\partial \text{SOG}} \end{array}\right]}^{T}$$

$\sigma_{COG}$—the course over ground mean measurement error; $\sigma_{SOG}$—the speed over ground mean measurement error.

Knowing the covariance matrix of initial (the previous) position coordinates **P**(k) and the covariance matrix of the vector of growth coordinates **P**(k+1/1), taking into account the errors resulting from the operation of dead reckoning navigation, the covariance matrix of the coordinates of the current BAUV position can be determined

(10) P(k+1)=P(k)+P(k+1/1)

where

$$P\left(k+1\right)=\left[\begin{array}{cc} {\sigma_{x}}^{2} & \sigma_{xy}\\ \sigma_{yx} & {\sigma_{y}}^{2} \end{array}\right]$$

$${\sigma_{x}}^{2}=\Delta t^{2}\cdot {\left(SOG\left(k\right)\cdot \sigma_{COG}\cdot \cos\text{COG}\left(k\right)\right)}^{2}+\Delta t^{2}\cdot {\left(\sigma_{SOG}\cdot \sin\text{COG}\left(k\right)\right)}^{2}$$

$${\sigma_{y}}^{2}=\Delta t^{2}\cdot {\left(SOG\left(k\right)\cdot \sigma_{COG}\cdot \sin\text{COG}\left(k\right)\right)}^{2}+\Delta t^{2}\cdot {\left(\sigma_{SOG}\cdot \cos\text{COG}\left(k\right)\right)}^{2}$$

$$\sigma_{xy}=\sigma_{yx}=\Delta t^{2}\cdot \sin\left(2\cdot \text{COG}\left(k\right)\right)\cdot \left({\sigma_{SOG}}^{2}-{\left(SOG\cdot \sigma_{COG}\right)}^{2}\right)/2$$

Using this matrix, in turn, the mean error of coordinates can easily be calculated

(11) $$M_{xy}=\sqrt{{\sigma_{x}}^{2}+{\sigma_{y}}^{2}}$$

and the parameters of the mean error ellipse:

(12) $$a=\sqrt{\frac{1}{2}\cdot \left({\sigma_{x}}^{2}+{\sigma_{y}}^{2}+\sqrt{p}\right)}$$

(13) $$b=\sqrt{\frac{1}{2}\cdot \left({\sigma_{x}}^{2}+{\sigma_{y}}^{2}-\sqrt{p}\right)}$$

(14) $$\tau =\frac{1}{2}\;\text{arc tg}\frac{2\sigma_{xy}\;}{{\sigma_{x}}^{2}-{\sigma_{y}}^{2}}$$

where

$$p={\left({\sigma_{x}}^{2}-{\sigma_{y}}^{2}\right)}^{2}+4\cdot {\sigma_{xy}}^{2}$$

τ—direction angle of ellipse.

**Figure 5:**
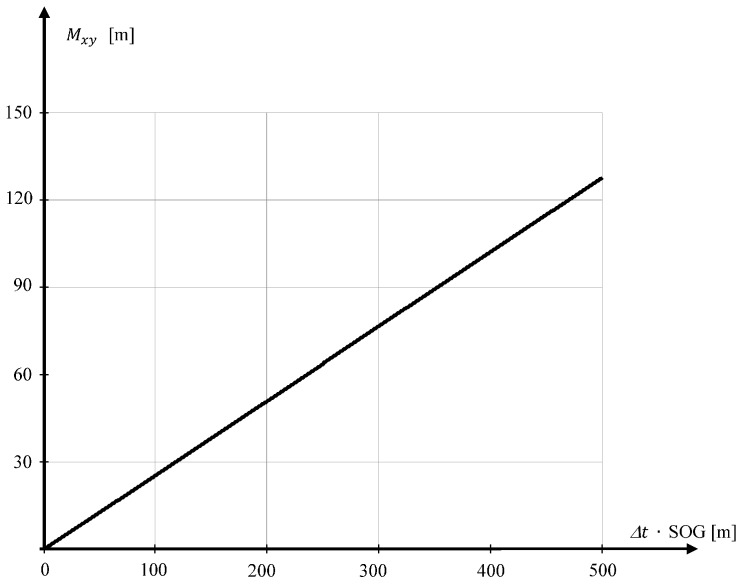
Mean error of coordinates as a function of distance covered (adopted $P\left(k=0\right)=\left[\begin{array}{cc} 0 & 0\\ 0 & 0 \end{array}\right]$, SOG = 1 m/s, COG = 90°).

Relying on the information provided by the manufacturer of log “ALIZE” that $\sigma_{SOG}=0.5$ kn and determined arbitrarily $\sigma_{COG}=3{}^{\circ}$ for INS “VN-200”, using Equations (10) and (11), a mean error graph of position coordinates determined using dead reckoning was prepared (Figure 5).

Figure 5 shows that the mean error of position coordinates increases linearly as a function of distance. Every hundred meters it increases constantly at 25.54 m, reaching a value of 127.7 m after traveling 500 m.

**Figure 6:**
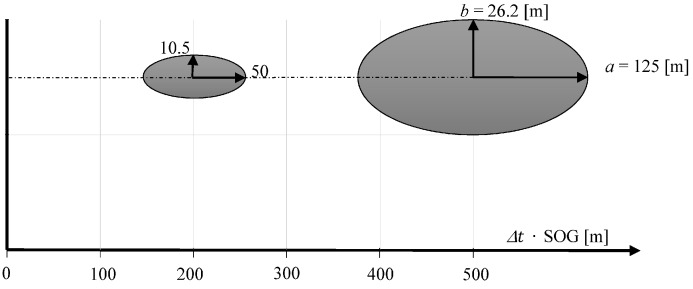
Change of parameters of mean error ellipse after covering a distance of 200 and 500 m.

Figure 6 shows the mean error ellipses of position coordinates determined using the dead reckoning method after traveling 200 and 500 m (their dependencies No. 12–13 have been calculated).

Figure 6 shows that the largest coordinate error occurs in the direction according to COG. The length of the axis b is equal to the length of the mean error ellipse of coordinates determined using USBL system (Figure 4). This is due to the fact that in both methods of determining the position coordinates the positioning line is a result of the direction measurement that is burdened with the same error value $\sigma_{COG\;}=\sigma_{\alpha }$.

## 4. The Genesis of the Problem

The choice between the USBL system and the method of dead reckoning based on the accuracy criterion is difficult. In some cases, the USBL system can be identified as being better (e.g., when measurements are carried out in small distances from the transceiver), while in others, the method of dead reckoning (e.g., immediately after updating coordinates by GNSS fixes). If a USBL system is selected, you give up additional COG and SOG. If you choose the DR method measurements r, α, β (range, yaw, pitch) are not taken into account. It is certain that skilful use of a greater number of measurements can improve their accuracy [29].

In the case of the NS BAUV, the different availability of measurements is assumed, i.e.,

- at regular intervals of time and with high frequency—the course and speed determined by navigation devices installed on the BAUV (not less than once per second),
- at irregular intervals and with low frequency—the direction and distance to the Transceiver of the appointed USBL system and transmitted by hydro-modem (less than once per second—because of the difficulties in carrying out USBL system measurements and sending the results of these measurements by hydro-modem caused interference in the propagation of acoustic waves in the water).

These parameters will then be used to determine the position coordinates using the extended Kalman filter (EKF).

In its action, the filter may combine the various measurements (e.g., the course with the distance to a navigation mark), carried out at different time intervals, taking into account their errors—adjusted dynamically by the matrix of weights. Thanks to this mechanism, measurements which are obviously erroneous (e.g., carried out in the USBL system, on the border of its operation) can be firmly suppressed, and accurate measurements amplified (e.g., SOG and COG determined in a short period of time from the moment of updating the coordinates by GNSS fixes).

However, will the coordinates obtained by the EKF method, a combination of parameter measurements of BAUV motion and the direction and distance of the USBL system, be more accurate than either the coordinate USBL system or the DR method in all cases?

The rest of this article presents a study on the positioning accuracy of BAUV by the EKF method compared to the USBL and DR positioning system, aimed at finding answers to the above question.

## 5. Description of Data Fusion Using the Extended Kalman Filter

Let us carry out a fusion of data using the extended Kalman filter combining COG(k) and SOG(k) measurements, changes in submergence Δz(k) carried out every one second with 3D coordinate system measurements of the USBL system, carried out at different time intervals (in some moments k+1).

Let us assume that the results of the USBL measurement system will create the so-called vector of observation, $z\left(k+1\right)={\left[\begin{array}{c} r\left(k+1\right)\;\alpha \left(k+1\right)\; \end{array}\beta \left(k+1\right)\right]}^{T}$, described by the function [30]:

(15) $$h\left(x\left(k+1\right),\nu \left(k+1\right)\right)=\left[\begin{array}{c} f_{r}\left(k+1\right)\\ f_{\alpha }\left(k+1\right)\\ f_{\beta }\left(k+1\right) \end{array}\right]=\left[\begin{array}{c} r\left(k+1\right)\\ \alpha \left(k+1\right)\\ \beta \left(k+1\right) \end{array}\right]+\left[\begin{array}{c} \nu_{r}\left(k+1\right)\\ \nu_{\alpha }\left(k+1\right)\\ \nu_{\beta }\left(k+1\right) \end{array}\right]$$

where

$$r\left(k+1\right)=\sqrt{{\left(x_{1}\left(k+1\right)-x\left(k+1\right)\right)}^{2}+{\left(y_{1}\left(k+1\right)-y\left(k+1\right)\right)}^{2}+{\left(z_{1}\left(k+1\right)-z\left(k+1\right)\right)}^{2}}$$

$$\alpha \left(k+1\right)=\text{arc tg}\frac{x_{1}\left(k+1\right)-x\left(k+1\right)}{y_{1}\left(k+1\right)-y\left(k+1\right)}$$

$$\beta \left(k+1\right)=-\text{ arc tg}\frac{z_{1}\left(k+1\right)-z\left(k+1\right)}{\sqrt{{\left(x_{1}\left(k+1\right)-x\left(k+1\right)\right)}^{2}+{\left(y_{1}\left(k+1\right)-y\left(k+1\right)\right)}^{2}}}$$

$\nu_{r}\left(k+1\right)$, $\nu_{\alpha }\left(k+1\right)$, $\nu_{\beta }\left(k+1\right)$—values of measurement errors (with zero mean normal distribution); x(k+1), y(k+1), z(k+1)—fixed coordinates of transceiver position; $x_{1}\left(k+1\right)$, $y_{1}\left(k+1\right)$, $z_{1}\left(k+1\right)$—variable coordinates of the transponder position (BAUV). And on the basis of COG(k), SOG(k) measurements and the submergence changes Δz(k), vector x of BAUV’s 3D position coordinates, using the function f(x(k),u(k),w(k)):

(16) $$x\left(k+1\right)\;=\left[\begin{array}{c} \begin{array}{c} f_{x}\left(k+1\right)\\ f_{y}\left(k+1\right)\\ f_{z}\left(k+1\right) \end{array} \end{array}\right]=\left[\begin{array}{c} \begin{array}{c} x\left(k\right)\\ y\left(k\right)\\ z\left(k\right) \end{array} \end{array}\right]+\left[\begin{array}{c} \begin{array}{c} \Delta t\left(k\right)\cdot \text{SOG}\left(k\right)\cdot \sin\text{COG}\left(k\right)\\ \Delta t\left(k\right)\cdot \text{SOG}\left(k\right)\cdot \cos\text{ COG}\left(k\right)\\ \Delta z\left(k\right) \end{array} \end{array}\right]+\left[\begin{array}{c} w_{x}\left(k\right)\\ w_{x}\left(k\right)\\ w_{z}\left(k\right) \end{array}\right]$$

In pursuing data fusion, based on functions h(x(k+1),0) i f(x(k),u(k),0) we calculate the estimated vector of coordinates $\hat{x}(k+1)$ and the covariance matrix P(k+1), using the following dependencies:

(17) $$\hat{x}{\left(k+1\right)}^{-}=f(x\left(k\right),u\left(k\right),0)$$

(18) $$P{\left(k+1\right)}^{-}=F\left(k+1\right)P\left(k\right)F{\left(k\;+\;1\right)}^{T}+Q\left(k\right)$$

(19) $$\tilde{y}(k+1)=z(k+1)-h(\hat{x}{\left(k+1\right)}^{-},0)$$

(20) $$S\left(k+1\right)(H\left(k+1\right)P{\left(k+1\right)}^{-}H{\left(k+1\right)}^{T}+R\left(k+1\right)$$

(21) $${K(k+1)=P(k+1)}^{-}H{\left(k+1\right)}^{T}\;S{\left(k+1\right)}^{-1}$$

(22) $$\hat{x}(k+1)=\hat{x}{\left(k+1\right)}^{-}+K(k+1)\tilde{y}(k+1)$$

(23) $$P(k+1)+\left(I-K(k+1)H(k+1)\right){P(k+1)}^{-}$$

where ${\hat{x}(k+1)}^{-}\;i\;P{\left(k+1\right)}^{-}$—estimated vector of coordinates of the BAUV position and its covariance matrix, determined a priori for the time of *k* + 1; $\hat{x}(k+1)\;i\;P(k+1)$—estimated vector of coordinates of the BAUV position and its covariance matrix, determined a posteriori for the time of *k* + 1, $F\left(k+1\right)=\left[\begin{array}{ccc} \frac{\partial f_{x}}{\partial x} & \frac{\partial f_{x}}{\partial y} & \frac{\partial f_{x}}{\partial z}\\ \frac{\partial f_{y}}{\partial x} & \frac{\partial f_{y}}{\partial y} & \frac{\partial f_{y}}{\partial z}\\ \frac{\partial f_{z}}{\partial x} & \frac{\partial f_{z}}{\partial y} & \frac{\partial f_{z}}{\partial z} \end{array}\right]=\left[\begin{array}{ccc} 1 & 0 & 0\\ 0 & 1 & 0\\ 0 & 0 & 1 \end{array}\right]$—the so-called matrix system is calculated as a function of the Jacobian matrix f(x(k),u(k),0), $Q\left(k\right)=\left[\begin{array}{ccc} {\sigma_{x}}^{2} & \sigma_{xy} & \sigma_{xz}\\ \sigma_{yx} & {\sigma_{y}}^{2} & \sigma_{yz}\\ \sigma_{zx} & \sigma_{zy} & {\sigma_{z}}^{2} \end{array}\right]=\left[\begin{array}{ccc} \frac{\partial f_{x}}{\partial COG} & \frac{\partial f_{x}}{\partial \text{SOG}} & \frac{\partial f_{x}}{\partial \Delta z}\\ \frac{\partial f_{y}}{\partial \text{COG}} & \frac{\partial f_{y}}{\partial \text{SOG}} & \frac{\partial f_{y}}{\partial \Delta z}\\ \frac{\partial f_{z}}{\partial \text{COG}} & \frac{\partial f_{z}}{\partial \text{SOG}} & \frac{\partial f_{z}}{\partial \Delta z} \end{array}\right]\left[\begin{array}{ccc} {\sigma_{COG}}^{2} & 0 & 0\\ 0 & {\sigma_{SOG}}^{2} & 0\\ 0 & 0 & {\sigma_{\Delta z}}^{2} \end{array}\right]{\left[\begin{array}{ccc} \frac{\partial f_{x}}{\partial COG} & \frac{\partial f_{x}}{\partial \text{SOG}} & \frac{\partial f_{x}}{\partial \Delta z}\\ \frac{\partial f_{y}}{\partial \text{COG}} & \frac{\partial f_{y}}{\partial \text{SOG}} & \frac{\partial f_{y}}{\partial \Delta z}\\ \frac{\partial f_{z}}{\partial \text{COG}} & \frac{\partial f_{z}}{\partial \text{SOG}} & \frac{\partial f_{z}}{\partial \Delta z} \end{array}\right]}^{T}$—matrix of noises of the state vector at the time of *k* (with adopted mean errors $\sigma_{COG}$, $\sigma_{SOG}$, $\sigma_{\Delta z}$ of COG and SOG measurements and changes in submergence),

$$\sigma_{xz}=\sigma_{yz}=\sigma_{zx}=\sigma_{zy}=0$$

$${\sigma_{z}}^{2}={\sigma_{\Delta z}}^{2}$$

P(k) —updated covariance matrix of the state vector used in subsequent time *k* + 1, $H\left(k+1\right)=\left[\begin{array}{ccc} \frac{\partial f_{r}}{\partial x} & \frac{\partial f_{r}}{\partial y} & \frac{\partial f_{r}}{\partial z}\\ \frac{\partial f_{\alpha }}{\partial x} & \frac{\partial f_{\alpha }}{\partial y} & \frac{\partial f_{\alpha }}{\partial z}\\ \frac{\partial f_{\beta }}{\partial x} & \frac{\partial f_{\beta }}{\partial y} & \frac{\partial f_{\beta }}{\partial z} \end{array}\right]=\left[\begin{array}{ccc} \frac{x_{1}\left(k+1\right)-x\left(k+1\right)}{\sqrt{l_{3}\left(k+1\right)}} & \frac{y_{1}\left(k+1\right)-y\left(k+1\right)}{\sqrt{l_{3}\left(k+1\right)}} & \frac{z_{1}\left(k+1\right)-z\left(k+1\right)}{\sqrt{l_{3}\left(k+1\right)}}\\ \frac{y_{1}\left(k+1\right)-y\left(k+1\right)}{l_{2}\left(k+1\right)} & -\frac{x_{1}\left(k+1\right)-x\left(k+1\right)}{l_{2}\left(k+1\right)} & 0\\ \frac{\left(x_{1}\left(k+1\right)-x\left(k+1\right)\right)\cdot \left(z_{1}\left(k+1\right)-z\left(k+1\right)\right)}{\sqrt{l_{2}\left(k+1\right)}\cdot l_{3}\left(k+1\right)} & \frac{\left(y_{1}\left(k+1\right)-y\left(k+1\right)\right)\cdot \left(z_{1}\left(k+1\right)-z\left(k+1\right)\right)}{\sqrt{l_{2}\left(k+1\right)}\;\cdot \;l_{3}\left(k+1\right)} & -\frac{\sqrt{l_{2}\left(k+1\right)}}{l_{3}\left(k+1\right)} \end{array}\right]$—Jacobian matrix with function h(x(k),0),

$$l_{3}\left(k+1\right)={\left(x_{1}\left(k+1\right)-x\left(k+1\right)\right)}^{2}+{\left(y_{1}\left(k+1\right)-y\left(k+1\right)\right)}^{2}+{\left(z_{1}\left(k+1\right)-z\left(k+1\right)\right)}^{2}$$

$$l_{2}\left(k+1\right)={\left(x_{1}\left(k+1\right)-x\left(k+1\right)\right)}^{2}+{\left(y_{1}\left(k+1\right)-y\left(k+1\right)\right)}^{2}$$

$R\left(k+1\right)=\;\left[\begin{array}{ccc} {\sigma_{r}}^{2} & 0 & 0\\ 0 & {\sigma_{\alpha }}^{2} & 0\\ 0 & 0 & {\sigma_{\beta }}^{2} \end{array}\right]$—matrix of noises of the observation vector in time of *k* + 1 (adopted mean measurement errors: $\sigma_{r}$—range, $\sigma_{\alpha }$—yaw, $\sigma_{\beta }$—pitch); I—identity matrix.

## 6. Research and Analysis of the Obtained Results

Let us now do an experiment to software simulation of the BAUV passage along the straight section with simultaneous:

- generating COG—measurements of INS carried out by “VN-200”, SOG—carried out with log “ALIZE” and Δz—carried out with a hydrostatic pressure sensor “S-20 WIKA”;
- generating by r, α, β measurements—carried out by the USBL “MicronNav System”;
- calculating the BAUV estimated coordinates of positions, parallel to the USBL system, with the method of dead reckoning and using the extended Kalman filter.

**Figure 7:**
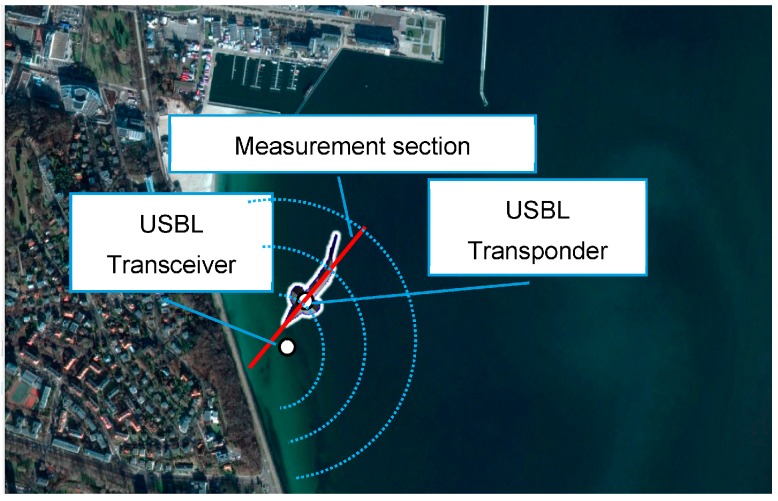
Measuring testing ground.

As a measuring testing ground, we assume a sea area around the USBL transponder system (Figure 7).

Let the BAUV swims near the USBL transceiver system (located at a depth of 6 m) overcoming the test section with COG= 220°, SOG= 1 m/s, submerged at z = 3 m. Meanwhile, τ=〈0 s, 600 s〉 simulated measurements will be carried out by the on-board BAUV and USBL system, i.e.,

- at fixed moments k+1 (every one second) $\text{COG}\left(k+1\right)=\;{\text{COG}}_{w}\left(k+1\right)+\Delta_{COG}$, $\text{SOG}\left(k+1\right)={\text{SOG}}_{w}\left(k+1\right)+\Delta_{SOG}$ and $\Delta z\left(k+1\right)=\;\Delta z_{w}\left(k+1\right)+\Delta_{\Delta z}$;
- at some moments k+1 (every 5, 20 and 100 s) $\begin{array}{c} r\left(k+1\right)=\; \end{array}r_{w}\left(k+1\right)+\Delta_{r}$, $\begin{array}{c} \alpha \left(k+1\right)=\alpha_{w}\left(k+1\right)+\Delta_{\alpha } \end{array}$, $\beta \left(k+1\right)=\beta_{w}\left(k+1\right)+\Delta_{\beta }$.

Each simulated value of the measurement is obtained by adding the reference measurement and the error (e.g., simulated $\text{COG}\left(k+1\right)=\;{\text{COG}}_{w}\left(k+1\right)+\Delta_{COG}$). Measurement error Δ will be treated as a random variable with uniform distribution, the value of which will be contained within three mean errors (3*σ*) of measurements made in the device (i.e., the probability of its occurrence will be at 99%).

Reference measurements of the USBL system $r_{w}\left(k+1\right)$, $\begin{array}{c} \alpha_{w}\left(k+1\right) \end{array}$, $\beta_{w}\left(k+1\right)$ will be determined relative to the reference positions consecutively occupied by the BAUV, which moves with undisturbed movement. On the other hand, the BAUV reference position will be dead reckoned (DR) on the basis of reference ${\text{COG}}_{w}\left(k+1\right)$, ${\text{SOG}}_{w}\left(k+1\right)$ and $\Delta z_{w}\left(k+1\right)$, which will be located exactly at the measuring section (see Figure 7).

For realigning the experiment for calculating the accepted mean error values close to the actual values (provided by the manufacturer and determined on the basis of their own—selected arbitrarily):

- $\sigma_{COG}=8{}^{\circ}$—for INS “VN-200”,
- $\sigma_{SOG}=0.5$ kn—for the electromagnetic log “ALIZE”,
- $\sigma_{\Delta z}=0.25\text{ m}$—for the hydrostatic pressure sensor “WIKA S-20”,
- $\sigma_{r}=0.2\text{ m}$, $\sigma_{\alpha }=\sigma_{\beta }=3{}^{\circ}$—for the USBL “MicronNav System” system.

The experiment will commence at the moment of k=0 and will be completed when the BAUV swims about 600 m. The extended Kalman filter will be “fine-tuned” at the start of the experiment and the coordinates of the initial BAUV position will be determined with a mean error of about 3 m (it is assumed that the coordinates were determined before submergence using GNSS) [31]. In the calculations, changes of COG, SOG, and Δz caused by the BAUV wave motion (swinging, on a sine curve) will be omitted.

### 6.1. Test No. 1

The test consisted of making three and then one hundred passages along the test section.

COG, SOG and Δz were measured every one second.

r, α, β were measured every 5 s.

#### First Passage

**Figure 8:**
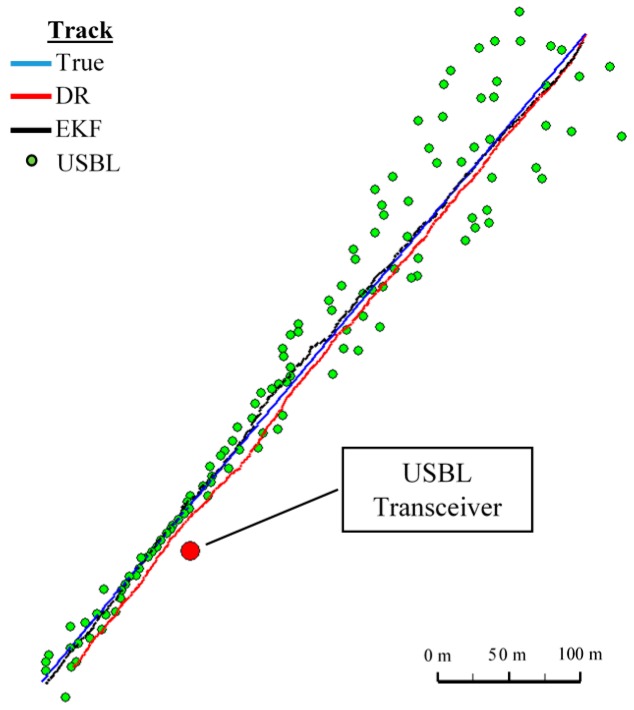
Determined routes (single passage).

**Figure 9:**
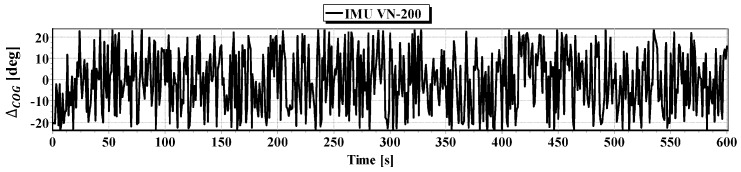
Graph of simulated measurement error $\Delta_{COG}$.

**Figure 10:**
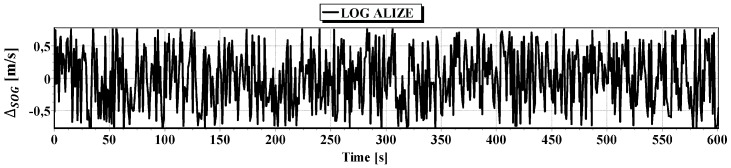
Graph of simulated measurement error $\Delta_{SOG}$.

**Figure 11:**
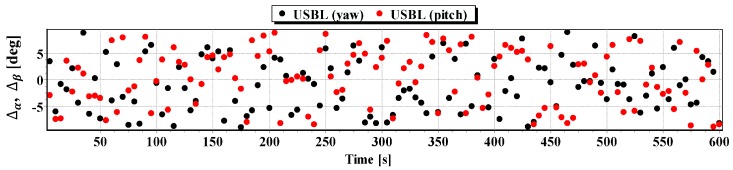
Graph of simulated measurement error $\Delta_{\alpha }$, $\Delta_{\beta }$.

**Figure 12:**
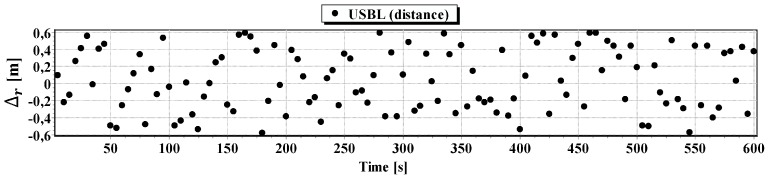
Graph of simulated measurement error $\Delta_{r}$.

In Figure 8 determined routs using different methods of positioning are presented. Figure 9, Figure 10, Figure 11 and Figure 12 present graphs depicting simulated measurement errors ($\Delta_{COG}$,$\;\Delta_{SOG}$,$\;\Delta_{r}$,$\;\Delta_{\alpha }$, $\Delta_{\beta }$) during the first passage of the test section (these graphs will not be displayed during the presentation of the results of further testing). Figure 9, Figure 10, Figure 11 and Figure 12 clearly show that the dispersion of the measurement errors are random and limited. The error value does not exceed 3σ fluctuating:

- $\Delta_{COG}$ in the range of 〈−15°, 15°〉,
- $\Delta_{SOG}$ in the range of 〈−0.8 m/s, 0.8 m/s〉,
- $\Delta_{\alpha }$, $\Delta_{\beta }$ in the range of 〈−9°, 9°〉,
- $\Delta_{r}$ in the range of 〈−0.6 m, 0.6 m〉.

**Figure 13:**
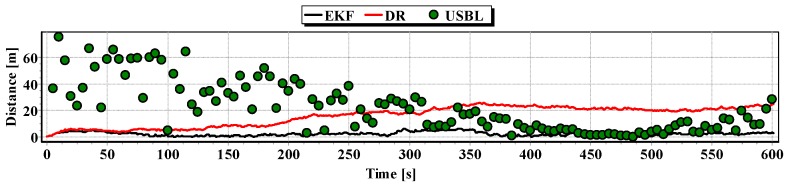
Graph of the reference position distance from the position estimated by EKF and DR methods and determined by the USBL system (single passage).

In Figure 13, for comparison, three graphs of the reference position distance from the position estimated by the extended Kalman filter and dead reckoning methods and determined by the USBL system are presented.

They show that the accuracy of the estimated position coordinates of EKF method is the same during the entire test duration. The accuracy of the estimated position coordinates using the DR method decreases as a function of time. On the other hand, the accuracy of the USBL position system grows in the time interval <400 s, 500 s>—it is then that the distance between the transceiver and the transponder does not exceed 50 m.

Table 1 shows the statistical parameters characterizing the accuracy of coordinates of all positions set at the test section.

The results in Table 1 clearly show that the best values of the statistical parameters were obtained by the EKF method.

**Figure 14:**
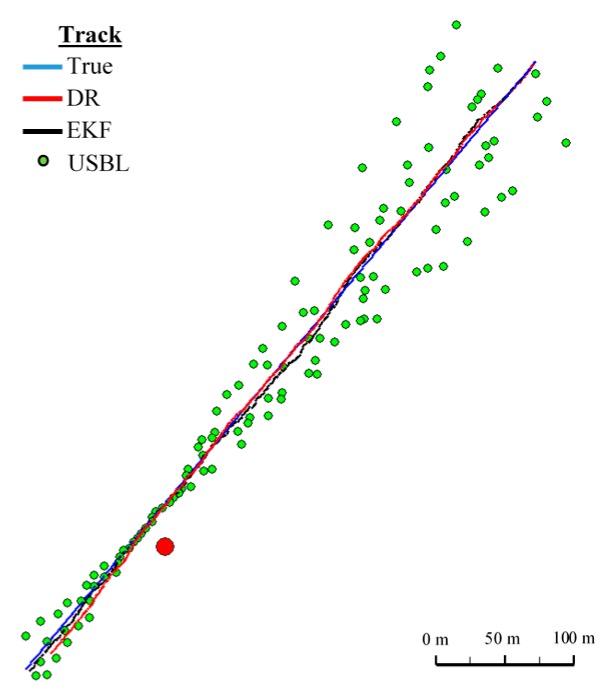
Determined routes (single passage).

**Figure 15:**
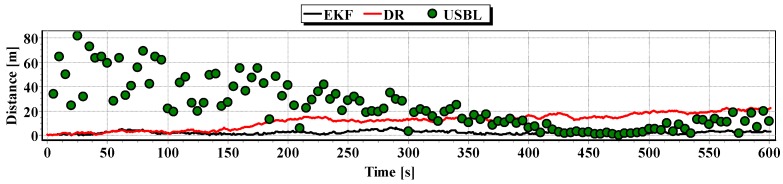
Graph of the reference position distance from the position estimated by EKF and DR methods and determined by the USBL system (single passage).

**Figure 16:**
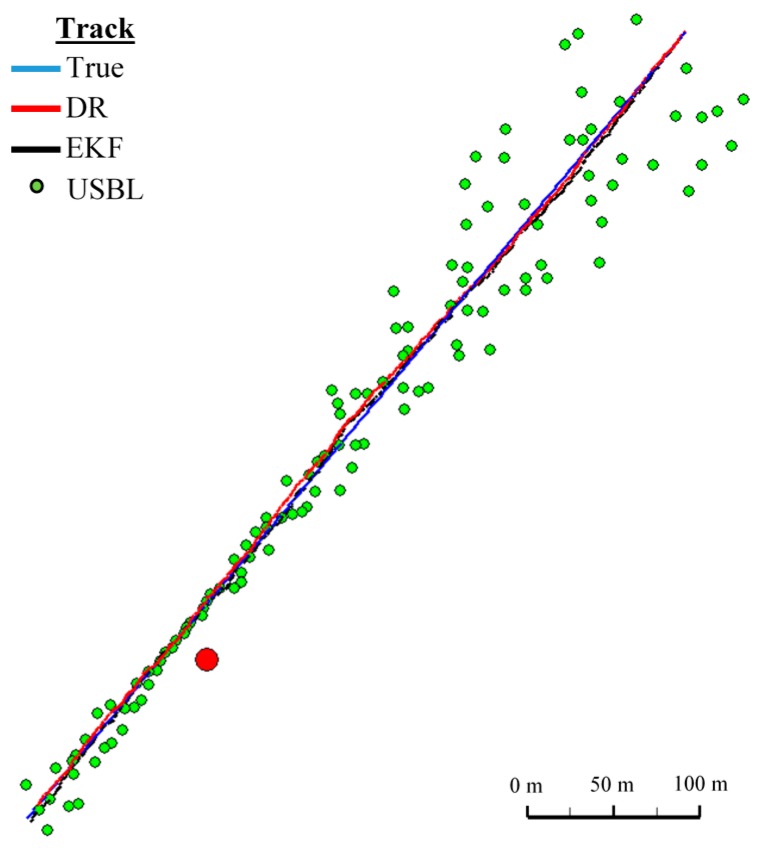
Determined route (single passage).

**Figure 17:**
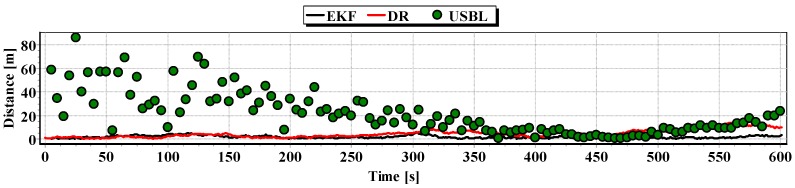
Graph of the reference position distance from the position estimated by EKF and DR methods and determined by the USBL system (single passage).

On the basis of simulation measurements during two successive passages along the test section, the following route graphs were prepared and the reference position distance from the estimated position (Figure 14, Figure 15, Figure 16 and Figure 17) as well as the statistical parameters (Table 2 and Table 3).

#### Second Passage

**Table 2:** Parameters of statistical positioning errors by EKF, DR, and USBL system (single passage).

Positioning Method	Minimum Distance to the Reference Position (m)	Maximum Distance to the Reference Position (m)	Average Distance to the Reference Position (m)
EKF	0.1	6.4	2.3
DR	0.1	22.6	11.8
USBL	0.2	81.6	23.6

#### Third Passage

**Table 3:** Parameters of statistical positioning errors by EKF, DR and USBL system (single passage).

Positioning Method	Minimum Distance to the Reference Position (m)	Maximum Distance to the Reference Position (m)	Average Distance to the Reference Position (m)
EKF	0.1	5.3	1.8
DR	0.2	14.0	4.7
USBL	0.7	86.1	21.6

On the basis of graphs analysis of Figure 14, Figure 15, Figure 16 and Figure 17, as well as statistical parameters from Table 2 and Table 3, it can be stated that the accuracy of estimated position coordinates using the EKF method is higher than those estimated using the DR method and determined by the USBL system. The reference position distance from the estimated one by EKF method does not exceed 7 m. Unfortunately, on the graphs it can be noticed that time intervals have occurred in which the reference position is further from the position estimated by EKF method, in comparison to the estimated position by DR method or determined using the USBL system. One can ask oneself a question here: how often do such time intervals occur? Generalized results of statistical analysis can answer that question. It is based on two additional indicators.

The former describes the average ξ distance to the reference position with estimated calculation on the basis of n distance ξ obtained for n passages along the test section at the same moment of k, in accordance with the formula:

(24) $$\xi (k)=\overset{n}{\underset{i=0}{\sum }}\zeta_{i}\left(k\right)$$

**Figure 18:**
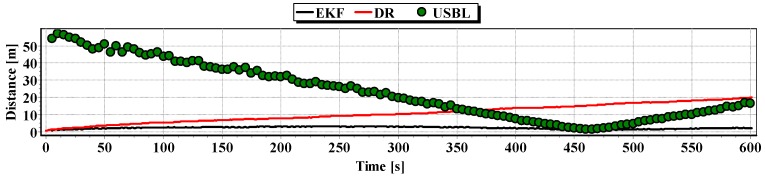
Graph of the reference position distance from the position estimated by EKF and DR methods and determined by the USBL system (one hundred passages).

**Figure 19:**
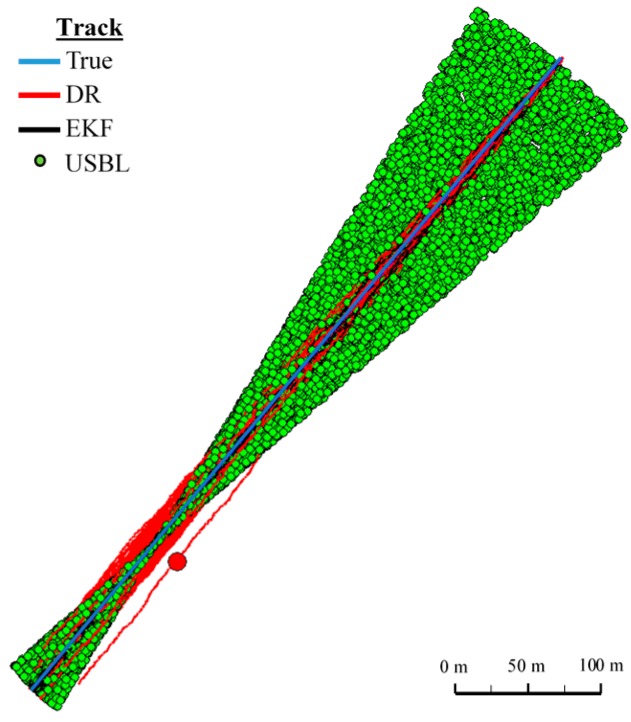
Determined routes (one hundred passages).

Figure 18 and Figure 19 presented the graph of the determined route, average distance to the reference position with the estimated, established on the basis of a hundred distance measurements carried out at the same moment, on each of the hundred BAUV passages of the test section.

#### One Hundred Passages

Generalizing the results of test No. 1, based on Figure 19, it can be stated that the average value of the reference position distance from the estimated position is constant in the case of EKF, it increases linearly for DR and decreases and then increases linearly in the case of the USBL system.

**Figure 20:**
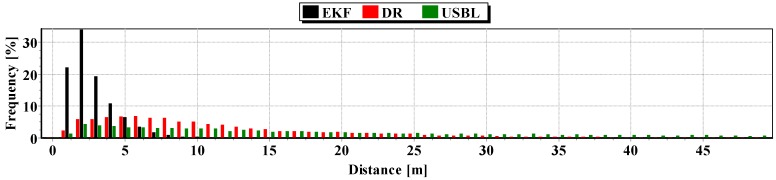
Histogram of the reference position distance from the position estimated by EKF and DR methods and determined by the USBL system (one hundred passages).

The second indicator to the generalized analysis is based on the histogram showing the frequency of the same distance to the reference position from the estimated position. The histogram is presented in Figure 20.

It results from the histogram that a two meter distance from the estimated position by EKF occurred most often. In addition, as a function of the distance to 5 m, EKF histogram columns occur most often. DR and USBL histograms are flattened as a function of distance. This proves that the incidence of very scattered distance values (up to 15 m) is highly probable. The statistical parameters of the positioning errors using EKF, DR methods and the USBL system are presented in Table 4.

The results in Table 4 clearly show that the best values of the statistical parameters were obtained by the EKF method.

### 6.2. Test No. 2

The test depended on carrying out three, followed by a hundred passages in the test section. COG, SOG and Δz were measured every one second. r, α, β were measured every 20 s.

#### First Passage

The statistical parameters of the positioning errors using EKF, DR methods and the USBL system for single passage are presented in Table 5 (for first passage) in Table 6 (for second passage) and in Table 7 (for third passage).

#### Second Passage

**Table 6:** Statistical parameters of positioning errors using EKF and DR methods and the USBL system (single passage).

Positioning Method	Minimum Distance to the Reference Position (m)	Maximum Distance to the Reference Position (m)	Average Distance to the Reference Position (m)
EKF	0.1	7.5	2.5
DR	0.1	26.7	9.7
USBL	1.4	64.3	21.0

#### Third Passage

**Figure 21:**
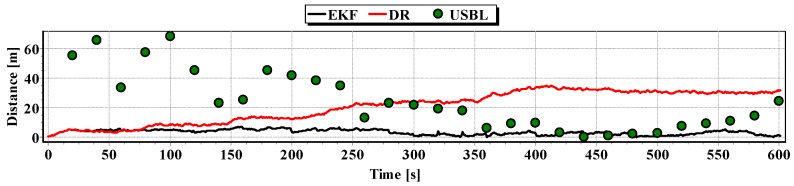
Graph of the reference position distance from the position estimated by EKF and DR methods and determined by the USBL system (single passage).

**Figure 22:**
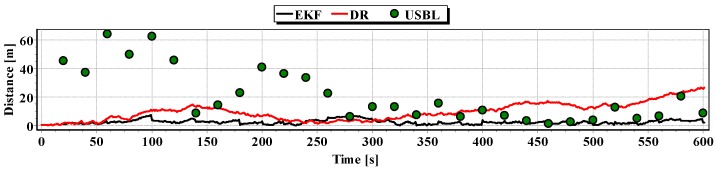
Graph distancing the reference position from the position determined by EKF and DR methods of the USBL system (single passage).

**Figure 23:**
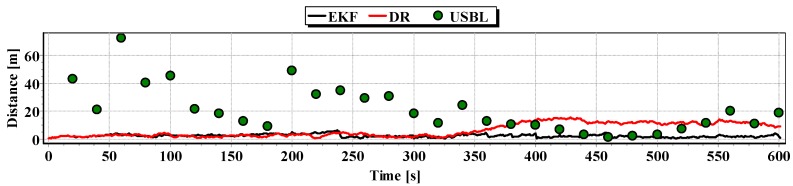
Graph of the reference position distance from the position estimated by EKF and DR methods and determined by the USBL system (single passage).

The analysis of Figure 21, Figure 22 and Figure 23 shows that the accuracy of the estimated position coordinates using EKF method improves almost every 20 s, when measurements from the USBL system are included in the calculations. Even if the position determined by the USBL system is significantly distant from the reference position, the accuracy of the estimated position using EKF method increases. This may be due to the fact that EKF suppresses the significant and obviously erroneous measurement direction of $\begin{array}{c} \alpha \left(k+1\right) \end{array}$, β(k+1) and reinforces the accurate measurement of the distance of r(k+1).

On the basis of analysis of the graphs in Figure 21, Figure 22 and Figure 23, and the statistical parameters from Table 6 and Table 7, it can be concluded that the accuracy of the estimated position coordinates using EKF method is greater than the estimated ones using DR method and determined by USBL system. The maximum reference position distance from the estimated ones using EKF method does not exceed 8 m. However, as in test No. 1, it can be seen on the graphs that there were intervals in which the reference position is more distant from the estimated position by the EKF method, compared to the estimated position by the DR method or that determined by the USBL system.

**Table 7:** Statistical parameters of positioning errors using EKF and DR methods and the USBL system (single passage).

Positioning Method	Minimum Distance to the Reference Position (m)	Maximum Distance to the Reference Position (m)	Average Distance to the Reference Position (m)
EKF	0.3	6.1	2.2
DR	0.3	15.3	6.2
USBL	1.3	72.6	21.1

#### One Hundred Passages

**Figure 24:**
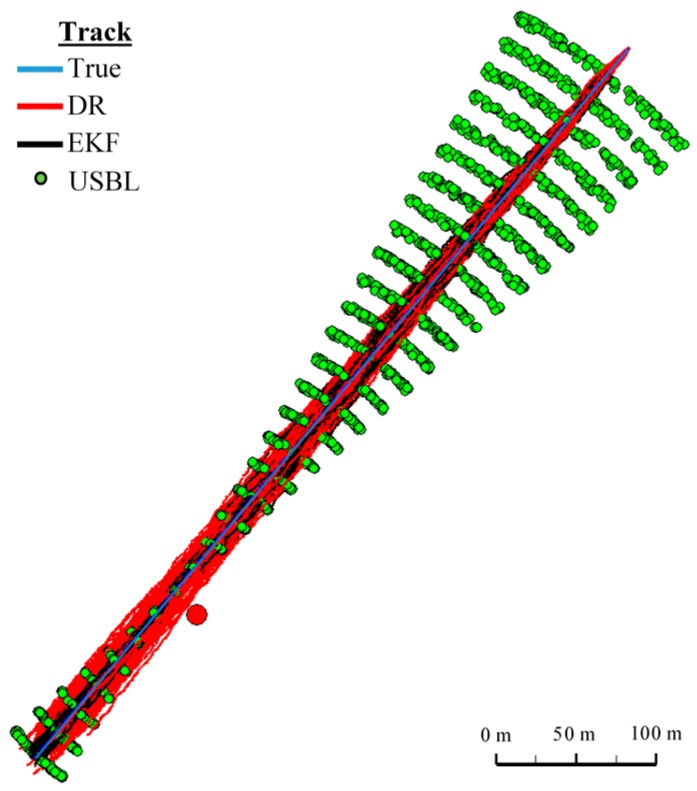
Determined route (one hundred passages).

**Figure 25:**
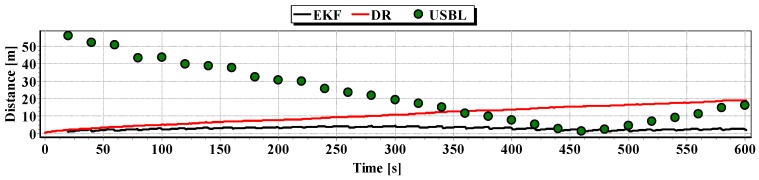
Graph of the reference position distance from the position estimated by EKF and DR methods and determined by the USBL system (hundred passages).

Figure 24 presents determined routs during one hundred passages using different methods of positioning. Graph of the reference position distance is presented in Figure 25.

**Figure 26:**
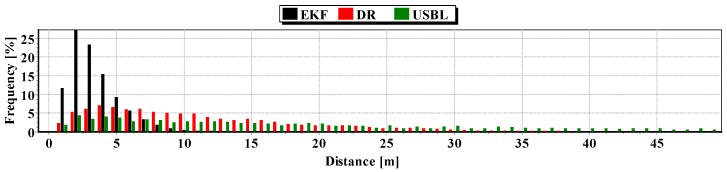
Histogram of the reference position distance from the position estimated by EKF and DR methods and determined by the USBL system (one hundred passages).

Generalizing the results of Test No. 2, they are similar to Test No. 1 based on the indicators presented in Figure 26 and Table 8.

### 6.3. Test No. 3

The test consisted of carrying out three, and then a hundred passages along the test section. COG, SOG and Δz were measured every one second. r, α, β were measured every 100 s. The statistical parameters of the positioning errors using EKF, DR methods and the USBL system for single passage are presented in Table 9 (for first passage) in Table 10 (for second passage) and in Table 11 (for third passage).

#### First Passage

**Table 9:** Statistical parameters of positioning errors using EKF, DR methods and the USBL system (single passage).

Positioning Method	Minimum Distance from the Reference Position (m)	Maximum Distance from the Reference Position (m)	Average Distance from the Reference Position (m)
EKF	0.1	11.6	4.0
DR	0.3	33.8	22.0
USBL	3.4	37.5	18.7

#### Second Passage

**Table 10:** Statistical parameters of positioning errors using EKF, DR methods and the USBL system (single passage).

Positioning Method	Minimum Distance from the Reference Position (m)	Maximum Distance from the Reference Position (m)	Average Distance from the Reference Position (m)
EKF	0.5	11.4	4.9
DR	0.5	28.7	12.0
USBL	7.6	20.0	14.0

#### Third Passage

**Table 11:** Statistical parameters of positioning errors using EKF, DR methods and the USBL system (single passage).

Positioning Method	Minimum Distance from the Reference Position (m)	Maximum Distance from the Reference Position (m)	Average Distance from the Reference Position (m)
EKF	0.2	9.1	4.0
DR	0.2	20.3	8.9
USBL	3.5	22.0	11.7

**Figure 27:**
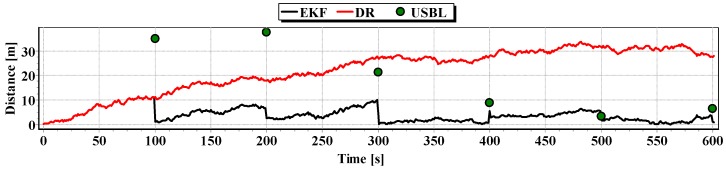
Graph of the reference position distance from the position estimated by EKF and DR methods and determined by the USBL system (single passage).

**Figure 28:**
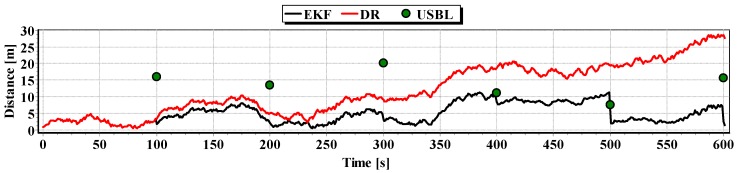
Graph of the reference position distance from the position estimated by EKF and DR methods and determined by the USBL system (single passage).

**Figure 29:**
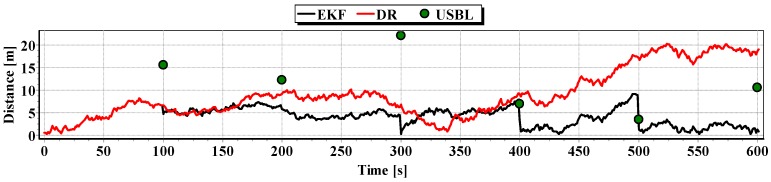
Graph of the reference position distance from the position estimated by EKF and DR methods and determined by the USBL system (single passage).

Graphs of the reference position distance from the position estimated by EKF and DR methods and determined by the USBL system for single passage are presented in Figure 27, Figure 28 and Figure 29. The analysis of these Figures shows that the accuracy of the estimated position coordinates using EKF method improves almost every 100 s, when measurements from the USBL system are included in the calculations. The accuracy of the estimated position coordinates using the EKF method is greater than the estimated ones using the DR method and determined by the USBL system. The reference position distance from the estimated ones using EKF method is slightly larger than in previous tests and reach about 11 m. This is due to the long period of estimating the position using EKF without taking measurements of the USBL system.

#### One Hundred Passages

**Figure 30:**
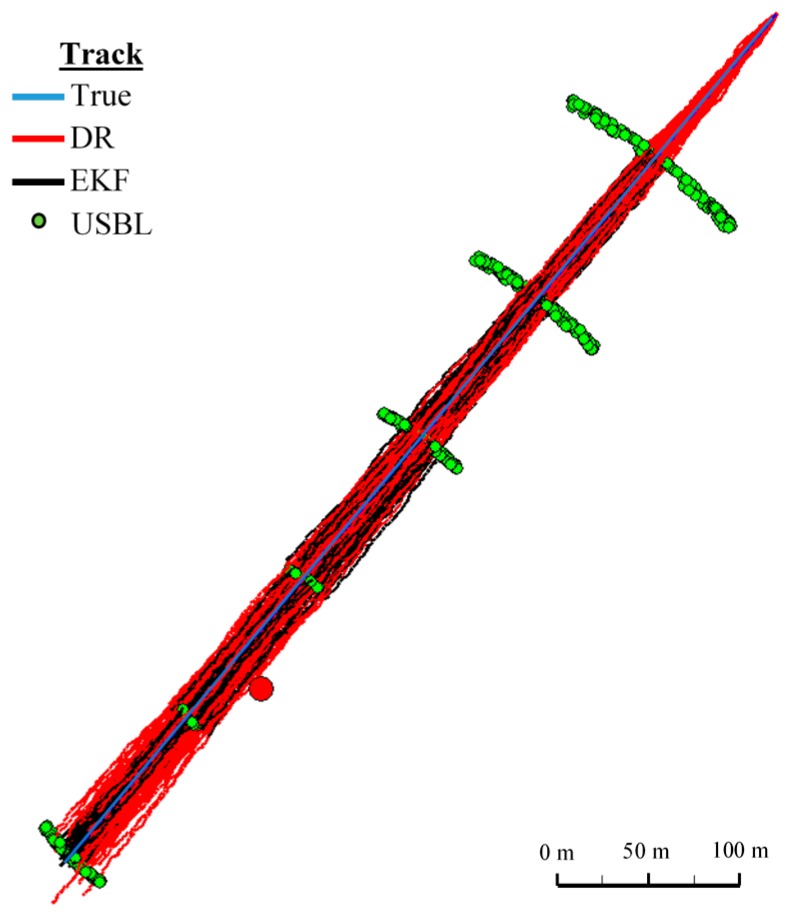
Determining the route (one hundred passages).

Figure 30 presents determined routs during one hundred passages using different methods of positioning. Statistical parameters of positioning errors using EKF, DR methods and the USBL system for these passages are presented in Table 12.

**Figure 31:**
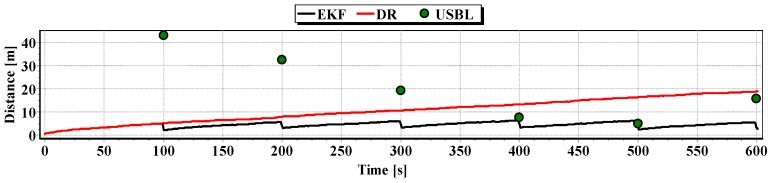
Graph of the reference position distance from the position estimated by EKF and DR methods and determined by the USBL system (one hundred passages).

**Figure 32:**
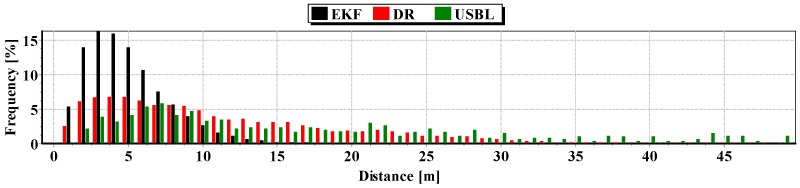
Histogram of the reference position distance from the position estimated by EKF and DR methods and determined by the USBL system (one hundred passages).

The generalized results of the Test No. 3, clearly show that the estimation of position coordinates using the EKF method gives the best results. In Figure 31, a marked improvement in the accuracy of the position can be seen when calculating additional measurements from the USBL system (at 100 s). The histogram in Figure 32 confirms the exact estimated position coordinates by the EKF method can be most frequently expected.

## 7. Conclusions

NS of the BAUV, which uses only the dead reckoning method based on the course and speed measurements, determines the position coordinates which error has quickly built up over time. In a study, after 500 s (corresponding to travelling 500 m) the average error of the position coordinates reached a value of 127.7 m (Figure 5).

In turn, the USBL system ensures high accuracy of determining the position coordinates, though only at short distances from the transceiver. In studies, at a distance of 500 m, the average error of determining the position coordinates will reach a value of 26.2 m (Figure 3).

Combining the EKF measurement method in the calculations carried out by devices installed on the BAUV (log, INS, hydrostatic pressure sensor) with distance and direction measurements carried out in the USBL system allows for obtaining the position coordinates significantly more accurate. This happens mainly because the position coordinates obtained are based on low accurate COG and SOG measurements and may be adjusted every now and then by more precise distance measurements of r carried out by the USBL system. This is confirmed by the full results of the three tests presented in the article.

The presented research concerned only method of BAUV positioning during undisturbed motion along the straight line. The obtained results are based only on simulated measurements. Further research will focus on tests in real environment. Sea currents and waving of sea will be taken into consideration. Furthermore, positioning accuracy of BAUV will be assessed during movement along composed trajectories including straight sections as well as curves lines.

## Figures and Tables

**Table 1:** Statistical parameters of positioning errors by EKF and DR methods and the USBL system (single passage).

Positioning Method	Minimum Distance to the Reference Position (m)	Maximum Distance to the Reference Position (m)	Average Distance to the Reference Position (m)
EKF	0.1	6.6	2.2
DR	0.7	25.8	15.9
USBL	0.3	75.2	22.6

**Table 4:** The statistical parameters of the positioning errors using EKF, DR methods and the USBL system (one hundred passages).

Positioning Method	Minimum Distance from the Reference Position (m)	Maximum Distance from the Reference Position (m)	Average Distance from the Reference Position (m)
EKF	0.6	3.3	2.2
DR	0.6	20.1	10.6
USBL	1.3	57.0	23.4

**Table 5:** Statistical parameters of positioning errors using EKF and DR methods and the USBL system (single passage).

Positioning Method	Minimum Distance to the Reference Position (m)	Maximum Distance to the Reference Position (m)	Average Distance to the Reference Position (m)
EKF	0.2	7.3	3.4
DR	0.7	34.9	10.9
USBL	0.4	68.0	24.3

**Table 8:** Statistical parameters of positioning errors using EKF, DR methods and the USBL system (single passage).

Positioning Method	Minimum Distance to the Reference Position (m)	Maximum Distance to the Reference Position (m)	Average Distance to the Reference Position (m)
EKF	0.7	4.4	2.9
DR	0.7	19.1	10.8
USBL	1.3	55.9	22.7

**Table 12:** Statistical parameters of positioning errors using EKF, DR methods and the USBL system (one hundred passages).

Positioning Method	Minimum Distance from the Reference Position (m)	Maximum Distance from the Reference Position (m)	Average Distance from the Reference Position (m)
EKF	0.6	6.4	4.4
DR	0.6	18.9	10.7
USBL	4.8	43.0	20.5
